# LncRNA AWPPH overexpression predicts the recurrence of periodontitis

**DOI:** 10.1042/BSR20190636

**Published:** 2019-07-26

**Authors:** Xiaofang Wang, Feng Ma, Peizeng Jia

**Affiliations:** 1 Beijing dental workers association Tian Xiu clinic Golden five-star Science Park, No.118 Zhongguancu’n East Road, Haidian District, Beijing City 102206, P.R. China; 2 Department of Orthodontics, Peking University School and Hospital of Stomatology, Beijing City 100081, P.R. China; 3 Department of Stomatology, School of Shandong University, Jinan City, Shandong Province 250012, P.R. China; 4 Department of Stomatology, Jinan Central Hospital, Shandong University, Jinan City, Shandong Province 250013, China

**Keywords:** lncRNA AWPPH, periodontitis, recurrence

## Abstract

Long non-coding RNA (LncRNA) AWPPH is a recently identified oncogenic lncRNA, while its role in other human diseases is still unknown. Blood samples from 80 periodontitis (periodontitis group) patients and 66 healthy controls (control group) who were admitted and treated by Peking University School and Hospital of Stomatology, expression levels of lncRNA AWPPH were detected by RT-PCR. In the present study, we showed that, before treatment, lncRNA AWPPH in plasma was up-regulated in periodontitis patients than in healthy controls. After treatment, expression levels of lncRNA AWPPH reduced significantly. Patients were followed up for 2 years to recorded recurrence. Compared with plasma levels of lncRNA AWPPH on the day of discharge, lncRNA AWPPH expression level increased significantly in patients with recurrence but not in patients without recurrence during follow-up. Based on Youden’s index, patients were divided into high and low lncRNA AWPPH groups according to its expression level on the day of discharge. It was observed that the recurrence rate of periodontitis is significantly higher in high lncRNA AWPPH group than in low lncRNA AWPPH group. LncRNA AWPPH overexpression predicts the recurrence of periodontitis.

## Introduction

Periodontitis is a type of severe gum infection that destroys the bone that supports teeth and damages soft tissue [1]. As a biofilm-induced chronic inflammatory disease, periodontitis causes adverse impact on systemic health [1]. Periodontitis affects ∼10% of population across the world, and it has become a major burden on public health [2,3]. Previous studies have shown that, various factors, such as low education level, male gender and diabetes are risk factors for periodontitis [4]. However, the molecular mechanism of the occurrence of periodontitis is still unclear [4]. Periodontitis patients are usually treated with systemic antibiotics and outcomes are generally satisfactory [5]. However, the misuse of antibiotics, such as improper drug selections and overdoses, are common, which in turn leads to other medical conditions [5].

Long non-coding RNAs, or lncRNAs, are a group of non-protein-coding RNAs composed of more than 200 nucleotides [6]. A growing body of literature has shown that lncRNAs are key players in human diseases [7,8]. During the development of periodontitis, a large set of lncRNAs were dysregulated and some of the differentially expressed lncRNAs have been proven to be involved in many critical aspects of the progression of periodontitis [9]. AWPPH is an lncRNA with oncogenic functions in several types of human cancers [10–12], while its involvement in other diseases is unknown. In the present study, we showed that lncRNA AWPPH was up-regulated in periodontitis and high lncRNA AWPPH level predicts the recurrence of periodontitis.

## Materials and methods

### Patients

Our study included 80 periodontitis (periodontitis group) patients and 66 healthy controls (control group) that were admitted and treated in Peking University School and Hospital of Stomatology from June 2012 to May 2014. Inclusion criteria: 1) patients who were diagnosed and treated for the first time, 2) patients with complete medical record; 3) patients completed treatment and 2-year follow-up in Peking University School and Hospital of Stomatology. Exclusion criteria: 1) patients who were treated within 3 months before admission; 2) patients failed to complete treatment in Peking University School and Hospital of Stomatology and were transferred to other hospitals; 3) patients who were lost during follow-up. The 80 periodontitis patients included 48 males and 32 females, with an age range of 38–62 years and a mean age of 48.3 ± 4.8 years. The control group included 39 males and 27 females, with an age range of 36–66 years and a mean age of 49.1 ± 5.1 years. Ethic Committee of Peking University School and Hospital of Stomatology approved the present study. All participants signed informed consent. See Table 1 for basic information of two groups of participants.

**Table 1:** Basic information of two groups of participants

	Periodontitis	Controls
Cases (*n*)	80	66
BMI	21.3 ± 1.1	21.4 ± 0.9
Smoking		
Yes	35	27
No	55	39
Drinking		
Yes	44	38
No	36	28
Gender		
Male	48	39
Female	32	27
Age range (years)	38–62	36–66
Mean age (years)	48.3 ± 4.8	49.1 ± 5.1

### Treatment

All patients received scaling, root planing and the use of both topical and oral antibiotics to control bacterial infection. Topical antibiotics were used by inserting antibiotics-containing gels into space between gums and teeth or into pockets after deep cleaning. Patients were discharged after all operations performed and inflammation was controlled.

### Follow-up

All patients completed treatment in Peking University School and Hospital of Stomatology. After discharge, patients were followed up for 2 years and the recurrence of periodontitis was recorded. Patients failed to complete follow-up or patients who developed other diseases were excluded from the present study. Initial and recurrent periodontitis were diagnosed by: 1) Mouth examination to check tartar buildup, plaque and easy bleeding. 2) Measurement of the pocket depth of the groove, and pockets deeper than 4 mm suggest possible periodontitis. 3) Dental X-rays to find bone loss.

### Plasma RNA extraction and real-Time Quantitative PCR

Blood (5 ml) was extracted on the day of admission, on the day of discharge and on the day of recurrence in cases of recurrence or at the end of follow-up in cases of non-recurrence. Plasma was extracted from blood using conventional methods, followed by total RNA extractions using exoRNeasy Serum/Plasma Kit (QIAGEN). Following reverse transcription using Applied Biosystems™ High-Capacity cDNA Reverse Transcription Kit, PCR reaction systems were prepared suing Luna® Universal One-Step RT-qPCR Kit (E3005, NEB). Primers of lncRNA AWPPH and endogenous control GAPDH used in PCR reactions were designed and synthesized by Sangon (Shanghai, China). Thermal reaction conditions were: 1 min at 95°C, followed by 10 s at 95°C and 25 s at 55.5°C for four cycles. Expression levels of lncRNA AWPPH were normalized to GAPDH using 2^−∆∆CT^ method. Primer sequences were: AWPPH forward: 5′-CTGGATGGTCGCTGCTTTTTA-3′ and reverse: 5′-AGGGGGATGAGTCGTGATTT-3′; GAPDH forward: 5′-GAAGGTGAAGGTCGGAGTC-3′ and reverse: 5′-GAAGATGGTGATGGGATTTC-3′.

### Statistical analysis

Experiments were performed three times. Comparisons between two groups were performed by unpaired *t* test. Comparisons within a group were performed by paired *t* test. Comparisons among three groups were performed by one-way ANOVA followed by Tukey’s test. Comparisons of recurrence rates were performed by χ^2^ test. All statistical analyses were performed using GraphPad Prism 6 software. Differences were statistically significant when *P*<0.05.

## Results

### LncRNA AWPPH in plasma was up-regulated in periodontitis patients than in healthy controls

**Figure 1:**
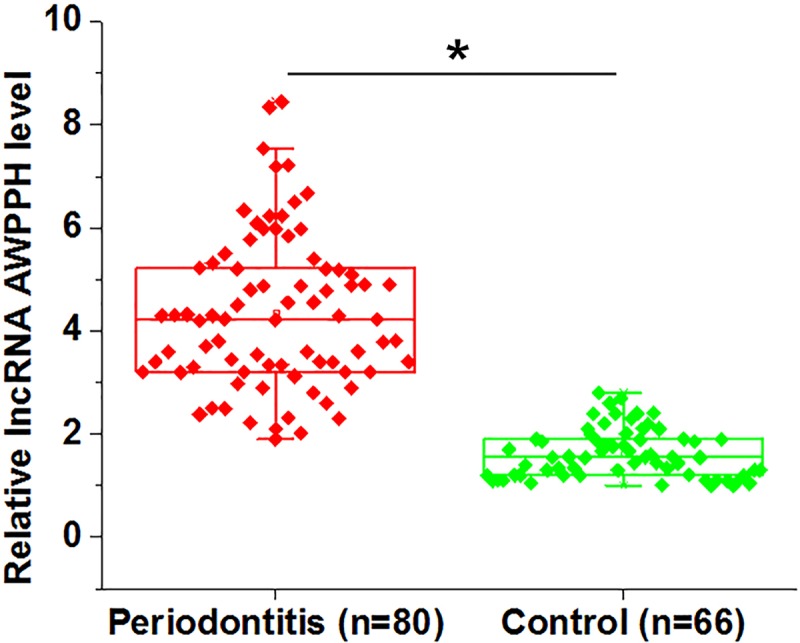
LncRNA AWPPH in plasma was up-regulated in periodontitis patients than in healthy controls Comparison of RT-qPCR results by unpaired *t* test showed that, compared with healthy controls, expression levels of lncRNA AWPPH in plasma were significantly increased in periodontitis patients (*, *P*<0.05). Data were presented by five lines, from lower to upper: minimum, lower 25%, median, upper 25% and maximum.

Before treatment, expression of lncRNA AWPPH in plasma of periodontitis patients and healthy controls was detected by real-Time Quantitative PCR (RT-qPCR). Compared with healthy controls, expression levels of lncRNA AWPPH in plasma were significantly increased in periodontitis patients (Figure 1, *P*<0.05).

### Expression levels of lncRNA AWPPH reduced significantly after treatment

**Figure 2:**
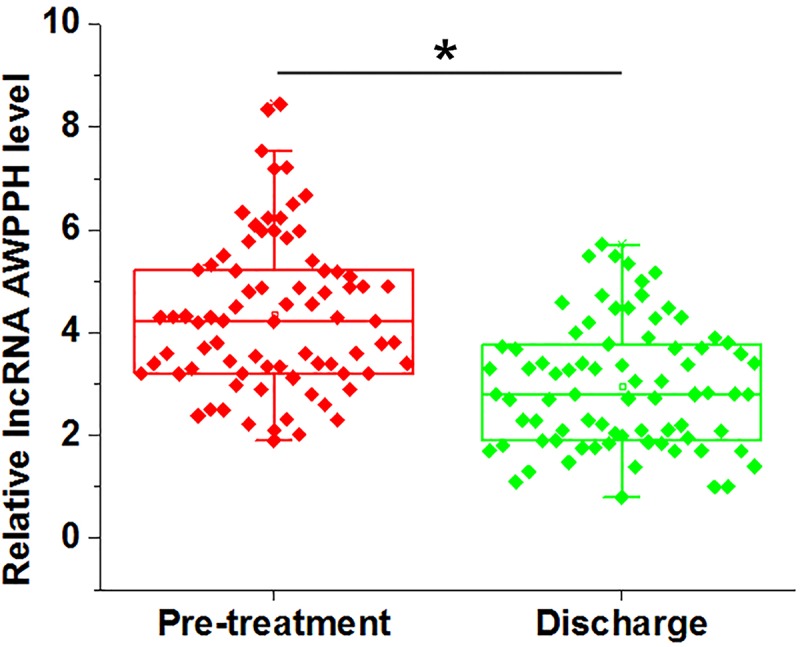
Expression levels of lncRNA AWPPH reduced significantly after treatment Comparison of RT-qPCR results by paired *t* test showed that, compared with pre-treatment level, expression levels of lncRNA AWPPH reduced significantly on the day of discharge. Data were presented by five lines, from lower to upper: minimum, lower 25%, median, upper 25% and maximum (*, *P*<0.05).

Expression of lncRNA AWPPH in plasma of periodontitis patients was also detected on the day of discharge by RT-qPCR. Compared with pre-treatment level, expression levels of lncRNA AWPPH reduced significantly on the day of discharge (Figure 2, *P*<0.05).

### AWPPH expression level increased significantly in patients with recurrence during follow-up

**Figure 3:**
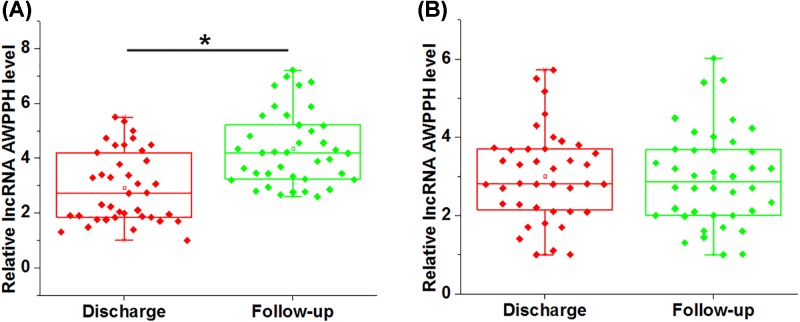
AWPPH expression level increased significantly in patients with recurrence during follow-up Comparison of RT-qPCR results by paired *t* test showed that, during follow-up, lncRNA AWPPH expression level increased significantly in patients with recurrence (**A**), but not in patients without recurrence during follow-up (**B**). Data were presented by five lines, from lower to upper: minimum, lower 25%, median, upper 25% and maximum, (*, *P*<0.05.)

During follow-up, recurrence was observed in 39 cases. Compared with plasma levels of lncRNA AWPPH on the day of discharge, lncRNA AWPPH expression level increased significantly in patients with recurrence (Figure 3A, *P*<0.05), but not in patients without recurrence during follow-up (Figure 3B).

### High lncRNA AWPPH level were significantly correlated with high recurrence rate

**Figure 4:**
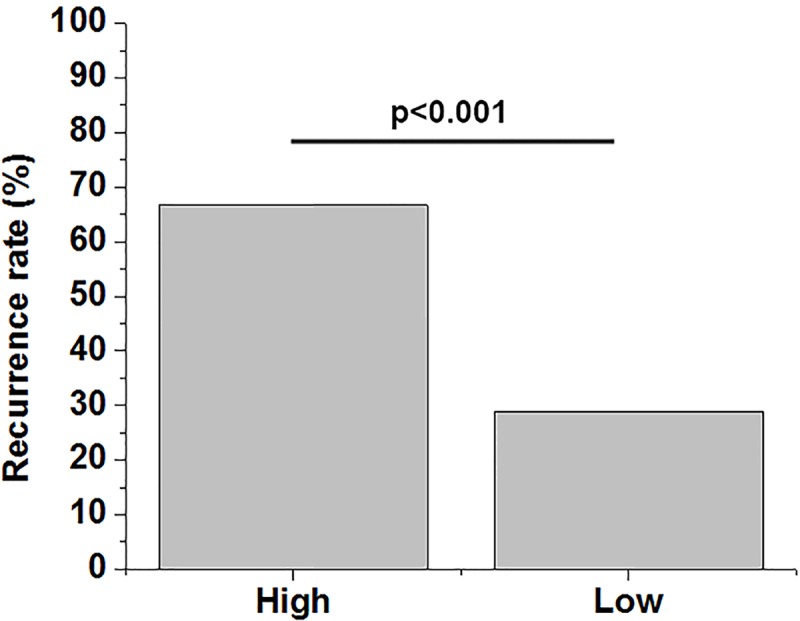
High lncRNA AWPPH level were significantly correlated with high recurrence rate Comparisons of recurrence rates were performed by χ^2^ test. Recurrence rate of periodontitis is significantly higher in high lncRNA AWPPH group than in low lncRNA AWPPH group. Data were presented by percentage (*, *P*<0.05).

Based on Youden’s index, patients were divided into high (*n*=42) and low (*n*=38) lncRNA AWPPH groups according to its expression level on the day of discharge. During follow-up, recurrence was observed in 39 cases, including 28 cases in high lncRNA AWPPH group, accounting for 66.7%, and 11 cases in low lncRNA AWPPH group, accounting for 28.9%. Recurrence rate of periodontitis is significantly higher in high lncRNA AWPPH group than in low lncRNA AWPPH group (Figure 4, *P*<0.001). It is worth noting that, there were 32 smokers included in the present study, included 18 cases in high lncRNA AWPPH group and 14 cases in low lncRNA AWPPH group. In addition, there were 41 alcohol drinker included in the present study, included 23 cases in high lncRNA AWPPH group and 19 cases in low lncRNA AWPPH group. Smoking and alcohol consumption showed no significant effects on lncRNA AWPPH expression.

## Discussion

LncRNA AWPPH is a well-characterized oncogene in several types of human cancers [10–12], while its involvement in other diseases is unknown. The key finding of the present study is that lncRNA AWPPH was up-regulated in periodontitis and the high lncRNA AWPPH expression level can be used to predict recurrence.

The development and progression of periodontitis globally affects the expression of lncRNAs [13]. However, studies on the clinical values of lncRNAs in periodontitis are rare. As an oncogenic lncRNA, AWPPH is up-regulated in different types of human malignancies [10–12]. Interestingly, lncRNA AWPPH in our study was also observed to be significantly up-regulated in periodontitis patients than in healthy controls, indicating the involvement of this lncRNA in periodontitis. After treatment, lncRNA AWPPH expression level was reduced, which further confirmed the involvement of lncRNA AWPPH and indicated that lncRNA AWPPH expression level may be used to guide treatment and reflect treatment outcomes.

Although treatment outcomes of periodontitis are generally satisfactory, high recurrence rate after discharge is also a big challenge in clinical practices [14–16]. At present, the prevention of recurrent periodontitis is still a key for the postoperative care of periodontitis [14–16]. In our study, we observed that lncRNA AWPPH expression level was increased only in patients with recurrence but not in patients without recurrence during follow-up. Therefore, the increase of lncRNA AWPPH may be used as an indicator of the recurrence of periodontitis and guide the postoperative care. In addition, our study also showed that high lncRNA AWPPH level is closely correlated with the recurrence. Our study only investigated the expression patterns of lncRNA AWPPH in periodontitis and explored the clinical values. However, the molecular mechanism of the role of lncRNA AWPPH in periodontitis is still unknown. Therefore, our future studies will aim to elucidate the mechanism of the actions of lncRNA AWPPH in periodontitis.

It is known that miR-124 [17] and miR-146a [18] play pivotal roles in periodontitis. We predicted the interactions between AWPPH and miR-124/miR-146a using IntaRNA. We found that AWPPH can form based parings with miR-124 and miR-146a (data not shown). Therefore, AWPPH may interact with these two miRNAs (such as sponging the miRNAs) to participate in periodontitis.

In conclusion, lncRNA AWPPH was up-regulated in periodontitis and high levels of lncRNA AWPPH predict postoperative recurrence of periodontitis.

## Abbreviations

GAPDH: glyceraldehyde-3-phosphate dehydrogenase
RT-qPCR: real-time quantitative PCR
